# The incorporation of the 3HP regimen for tuberculosis preventive treatment in the Brazilian health system: a secondary-database nationwide analysis

**DOI:** 10.3389/fmed.2023.1289298

**Published:** 2024-01-05

**Authors:** Luiz Villarinho Pereira Mendes, Anete Trajman, Mônica Rodrigues Campos, Marilena Cordeiro Dias Villela Correa, Claudia Garcia Serpa Osorio-de-Castro

**Affiliations:** ^1^Sergio Arouca National School of Public Health, Oswaldo Cruz Foundation, Rio de Janeiro, Brazil; ^2^Federal University of Rio de Janeiro, Rio de Janeiro, Brazil; ^3^Institute of Social Medicine of State University of Rio de Janeiro (IMS-UERJ), Rio de Janeiro, Brazil

**Keywords:** Brazil, tuberculosis, latent tuberculosis, latent tuberculosis infection, drug utilization studies, Rifapentine, isoniazid, public health

## Abstract

**Introduction:**

The recommendation of rifampin-based shorter - and safer – regimens for tuberculosis preventive treatment (TPT) is progressively replacing monotherapy with isoniazid by different countries. The Brazilian Ministry of Health (MoH) approved the incorporation of the Rifapentine + isoniazid regimen (3HP) at the end of 2020, with free distribution in the Brazilian Unified Health System (SUS) started from the last quarter of 2021. The objectives were to describe the implementation of the IL-TB System (Information System of TPT Notification) and uptake of Rifapentine + isoniazid (3HP) and Isoniazid (6H or 9H) in Brazil.

**Methods:**

A quantitative observational and descriptive was performed using the IL-TB National System as the main data source, from January 2018 to December 2022.

**Results and discussion:**

There was a steady increase of the number of TPT prescription quarterly throughout the period, which reflects the implementation of the system itself and the progressive adherence of the health system to the non-compulsory notification of new TPT. The substitution of isoniazid (6H or 9H) by 3HP is progressing. The 3HP regimen represented less than 4% of the total administered by the end of 2021, reaching around 30% in the second half of 2022 and 40% in the last quarters of 2022. The study points not only to the need to expand TPT in the country, but also to accelerate 3HP uptake and to encourage the municipalities to notify to the IL-TB system, since there is still a high level of underreporting.

## 1 Introduction

The recommendation of rifampin-based shorter - and safer – regimens for tuberculosis preventive treatment (TPT) is progressively replacing monotherapy with isoniazid by different countries based on society’s guidelines and World Health Organization (WHO)-recommendations (1–3). Regimens vary concerning the targeted populations and based on National Tuberculosis Control Program (NTCP)’ Choices: (i) 3 months of weekly doses of rifapentine and isoniazid (3HP) or 1 month of daily doses of the same combination (1HP); (ii) 3 months of daily doses of rifampicin and isoniazid (3RH) and (iii) 4 months of daily doses of rifampicin (4R). The WHO points to its advantages, mainly in terms of patient compliance and the reduced number of undesirable side-effects, resulting in better therapeutic adherence (2). Systematic reviews of clinical trials confirm its benefits: while 3HP present the best adherence, 4R is the safest regimen in adults (fewer side-effects) (4–7). However, little is known about how countries incorporate newer TPT regimens.

Brazil is a medium-high-income country with a high burden of TB and TB/HIV (8). In Brazil, TPT with isoniazid monotherapy is recommended since the 1980s for people living with HIV (PLWH) and contacts under 16 years of age. In 2010, TPT indication was expanded to contacts of any age. Since 2018, three TPT regimens were available in the country, all free of charge: 4R and 6 or 9 months of isoniazid (6H or 9H). 4R was the regimen of choice for children (≤ 10 years of age), older people (≥ 50 years of age) and those with chronic liver diseases. The choice between 6H and 9H was according to patients’ and health providers’ preference. 6H is the most commonly used monotherapy. Isoniazid is produced locally by the Brazilian Ministry of Health public pharmaceutical laboratories (3). The Brazilian Ministry of Health (MoH) approved the incorporation of the 3HP regimen at the end of 2020 (9), with free distribution in the Brazilian Unified Health System (SUS) started from the last quarter of 2021 (10). Since then, 3HP is recommended as the first-choice regimen for eligible candidates over 10 years of age (3). The objectives of this study were to describe the implementation of the IL-TB System and uptake of Rifapentine (3HP) and Isoniazid (6H or 9H) in Brazil.

## 2 Materials and methods

### 2.1 Study design

This is a quantitative observational and descriptive study using the IL-TB National System as the main data source, from January 2018 to December 2022.

### 2.2 Data source

IL-TB is a non-compulsory TPT prescription and follow-up notification system developed by the MoH. The system was implemented in the country in 2018 after a successful pilot phase in 2016/2017. Currently, all but 2 of the 27 Brazilian states have joined the IL-TB System (8). Demographic patient information, indication for treatment, date of treatment initiation, regimen (although 6H and 9H are not indicated – just “isoniazid monotherapy) and treatment outcomes were extracted from IL-TB. The National System of Compulsory Notifiable Diseases (SINAN) was also used for analysis of TB incidence at the municipal level. Like other SUS computerized systems, information is consolidated within 3 months of notification.

### 2.3 Procedures and variables

After extraction, transformation and uploading of the final IL-TB-original dataset, linkage with the SINAN (municipal level) was carried out. The index used for linkage was the Brazilian Institute of Geography and Statistics (IBGE) municipality code.

A descriptive analysis on demographic, clinical and health unit variables was performed stratified by the 5 Brazilian regions and a temporal quarterly analysis of regimen prescription and follow-up as the utilization proxy was carried out to estimate treatment uptake into the health system during the entire period.

The linkage with the SINAN database permitted calculation of the percentage of municipalities for each state with TB notification that reported at least one TPT in the IL-TB system. This percentage was used as a proxy for the level of adherence to the system by the states. An index to evaluate the proportion of treated TB contacts notified in the IL-TB system against the demand estimated by SINAN ideally one TPT for each new susceptible TB case, according to expert consensus (11, 12) was calculated, which provided us with an estimate of how much of the potential demand for contacts’ TPT has actually been addressed from the entire period.

To characterize the uptake of the 3HP regimen, we analyzed the period between July 2021 and December 2022, by quarters, expressing the proportion of each regimen through time. A further detailing of utilization patterns was carried out by calculating the number of Defined Daily Doses (DDD) per isoniazid treatment (considering the 6H or 9H versus 3HP). This indicator was calculated using the listed isoniazid DDD (0.3 g) (13) and expressed as number of DDD per 1,000 inhabitants (DID). The estimated Brazilian population for 2021 and 2022 was provided by IBGE (14).

The number of rifapentine (3HP) and isoniazid (6H or 9H) treatments distributed to each region for every 100 notified cases of active tuberculosis was calculated to account for the true demand for treatments in each region.

Among the valid database fields, for every 270-dose treatment (9H), 3,180-dose treatments (6H) were prescribed. This proportion was imputed to 9H and 6H treatments, respectively, based on valid “doses taken” records and applied to the entire database to calculate the total number of DDD.

### 2.4 Ethical considerations

The database was provided by the MoH in accordance with Law No. 12527/2011 (the access to information act) that regulates the availability of public data for research purposes. The database was stratified at the individual level and fully anonymized, and granted with full authorization from the MoH. Ethical approval is not necessary for anonymized secondary data analysis in Brazil (15). Concerning data from SINAN, it’s fully publicly available at the MoH website (16), and with no sensitive information at the individual level.

## 3 Results

Between 2018 and 2022, 111,941 treatments were recorded in the IL-TB database. The majority of notifications occurred in the Southeast (56%), the most developed and populated region, with the highest TB incidence. The North (23%) (Amazonian) region was the second. There was little difference regarding sex, with the proportion of men in the different regions varying between 50% (Centre West) and 56% (North). Most treatments were administered to people who declared themselves as Black or Brown (56%), followed by White people (36%). Most resided in the same municipality as the treatment facility (96%), 98% of treatments were new “cases” (first TPT), followed by a small percentage of re-entry after loss to follow up (1%) (Table 1).

**Table 1:** Sociodemographic and clinical characteristics of the individuals in TPT.

	Midwest (*N* = 4,042)		Northeast (*N* = 5,785)		North (25351)		South-East (62738)		South (14025)		Brazil (*N* = 111,941)	
	*N*	%	*N*	%	N	%	*N*	%	*N*	%	*N*	%
Sociodemographic variables												
Sex												
Female	2033	50%	3,268	56%	13,479	53%	33,024	53%	7,681	55%	59,485	53%
Male	2009	50%	2,517	44%	11,872	47%	29,714	47%	6,344	45%	52,456	47%
Race (self reported)												
Yellow	43	1%	24	0%	313	1%	434	1%	72	1%	886	1%
White	1,216	30%	568	10%	3,828	15%	24,965	40%	9,500	68%	40,077	36%
Ignored	71	2%	351	6%	1,199	5%	4,802	8%	1,000	7%	7,423	7%
Indigenous	322	8%	37	1%	459	2%	150	0%	43	0%	1,011	1%
Browns	2057	51%	3,603	62%	17,828	70%	24,765	39%	2,412	17%	50,665	45%
Black	333	8%	1,202	21%	1724	7%	7,622	12%	998	7%	11,879	11%
Does the individual live in the same municipality as the healthcare facilities?												
No	240	6%	210	4%	1742	7%	2061	3%	297	2%	4,550	4%
Yes	3,802	94%	5,575	96%	23,609	93%	60,677	97%	13,728	98%	107,391	96%
Clinical variables												
TB contact												
Ignored	129	3%	202	3%	1,391	5%	2,637	4%	317	2%	4,676	4%
No	1,337	33%	1,517	26%	7,076	28%	17,670	28%	4,309	31%	31,909	29%
Do not know	737	18%	462	8%	2,310	9%	4,880	8%	1,164	8%	9,553	9%
Yes	1839	45%	3,604	62%	14,574	57%	37,551	60%	8,235	59%	65,803	59%
Type of Entry												
New case	3,952	98%	5,708	99%	24,926	98%	61,227	98%	13,647	97%	109,460	98%
Re-entry after change of regimen	20	0%	4	0%	69	0%	304	0%	109	1%	506	0%
Re-entry after suspension due to clinical condition unfavorable to treatment	1	0%		0%	11	0%	46	0%	32	0%	90	0%
Reexposure	12	0%	24	0%	76	0%	287	0%	56	0%	455	0%
Re-entry after abandoning treatment	57	1%	49	1%	269	1%	874	1%	181	1%	1,430	1%
Discarded active TB?												
No	25	1%	27	0%	193	1%	453	1%	73	1%	771	1%
Yes	4,017	99%	5,758	100%	25,158	99%	62,285	99%	13,952	99%	111,170	99%
BCG?												
Ignored	275	7%	1,143	20%	3,447	14%	11,476	18%	3,285	23%	19,626	18%
No	287	7%	527	9%	2,136	8%	4,226	7%	856	6%	8,032	7%
Yes	3,480	86%	4,115	71%	19,768	78%	47,036	75%	9,884	70%	84,283	75%
Treatments												
Isoniazid (6H or 9H)	3,329	82%	5,196	90%	20,630	81%	55,007	88%	11,485	82%	95,647	85%
Rifampicin	262	6%	190	3%	1,313	5%	2,428	4%	1,086	8%	5,279	5%
Rifapentine + Isoniazid	451	11%	399	7%	3,408	13%	5,303	8%	1,454	10%	11,015	10%

January 2018–December 2022. Brazil.

**Figure 1:**
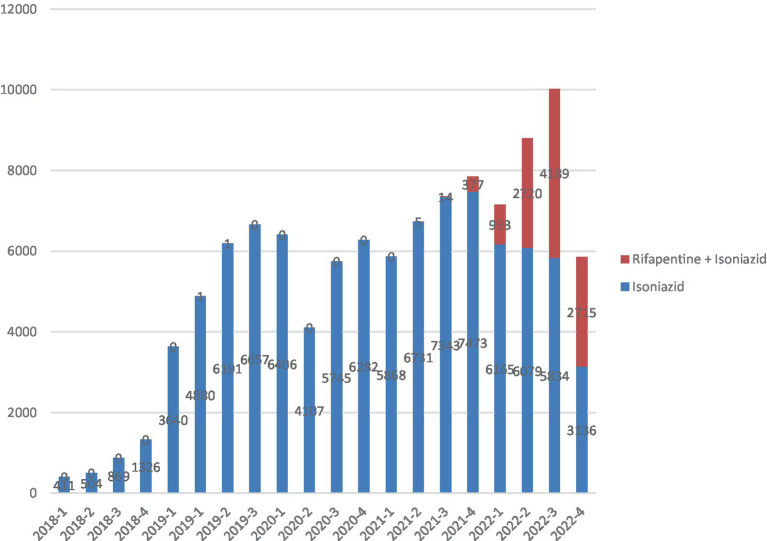
Total Isoniazid monotherapy (6H or 9H) or 3HP regimens reported in IL-TB (2018–2022).

There was a steady increase of the number of TPT prescription quarterly throughout the period, which reflects the implementation of the system itself and the progressive adherence of the health system to the non-compulsory notification of new TPT. Since the beginning of the implementation of the IL-TB system, monoisoniazid treatments increased (with some oscillation) until reaching 7,473 in the fourth quarter of 2021. From the first quarter of 2022 onwards, the number of monoisoniazid treatments started to decrease while 3HP progressively increased. Total number of treatments (3HP + 6H + 9H) increased, peaking at 10,000 in the third quarter of 2022. The data for the fourth quarter of 2022 may not be complete due to a possible delay in data reporting, as the dataset was exported in December of that year (Figure 1).

**Figure 2:**
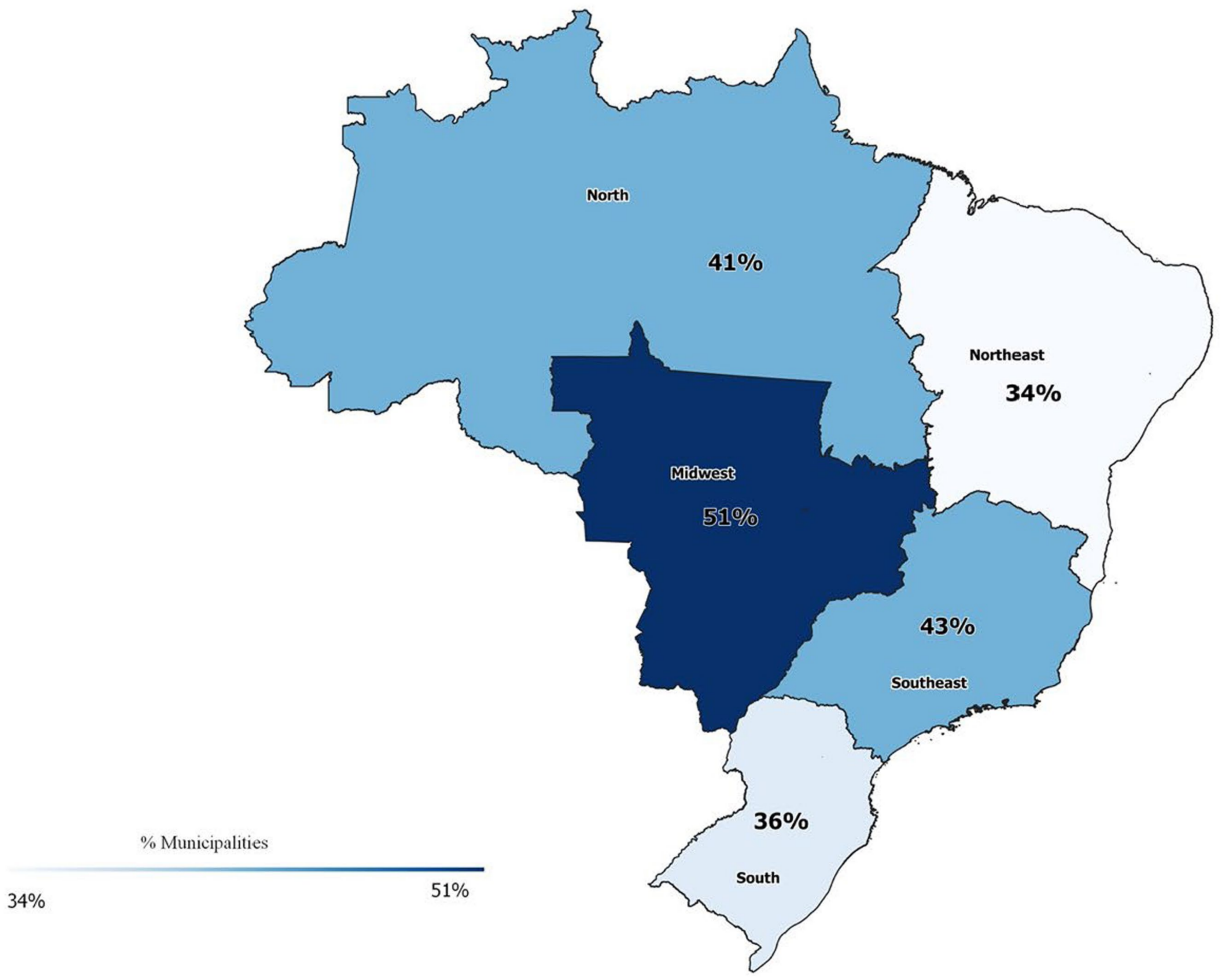
Percentage of Municipalities with SINAN TB notifications that reported at least one treatment for latent TB in the IL-TB system. Brazil, 2018–2022. *There is no data from the two states (Goiás in the Midwest and Santa Catarina in the South) that have not joined the IL-TB system.

58% of the Brazilian municipalities that notified TB in the last 5 years did not report any treatment of latent TB in the IL-TB system. The regions with the lowest percentage of municipalities notifying in the IL-TB were South (36%) and Northeast (34%). The regions with the highest percentage were Midwest (51%) and Southeast (43%) (Figure 2).

**Figure 3:**
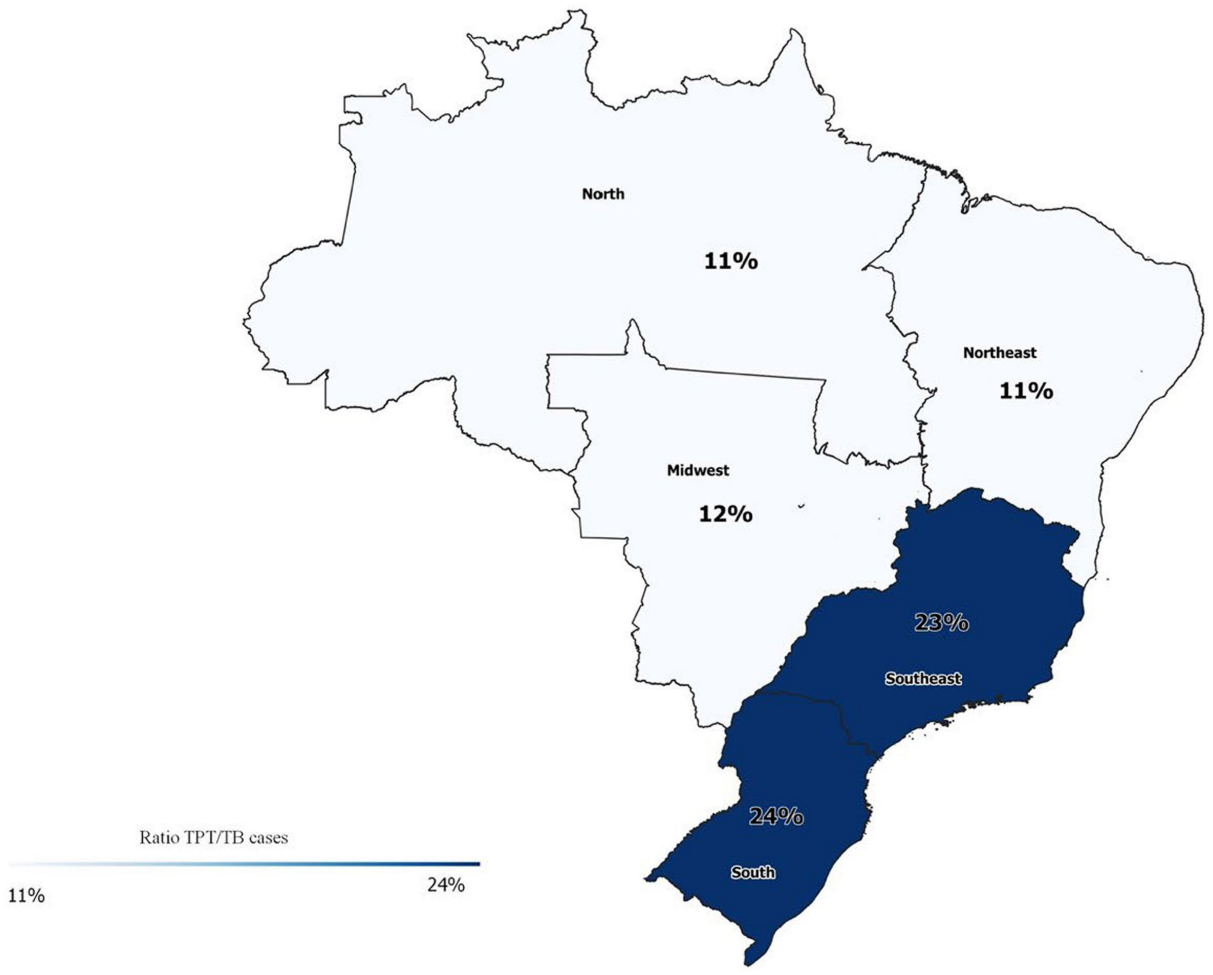
Ratio between new TPT in contacts (IL-TB, 2018–2022) and new TB cases (SINAN, 2018–2022). *There is no data from the two states (Goiás in the Midwest and Santa Catarina in the South) that have not joined the IL-TB system.

TPT in contacts (65,706 “cases” between 2018 and 2022) corresponds to 16% of the total active TB cases notified in the same period (n = 603,684). The regions with the highest rates were the South (24%) and Southeast (23%). The regions with the lowest rates were the North and Northeast (both with 11%) (Figure 3).

**Figure 4:**
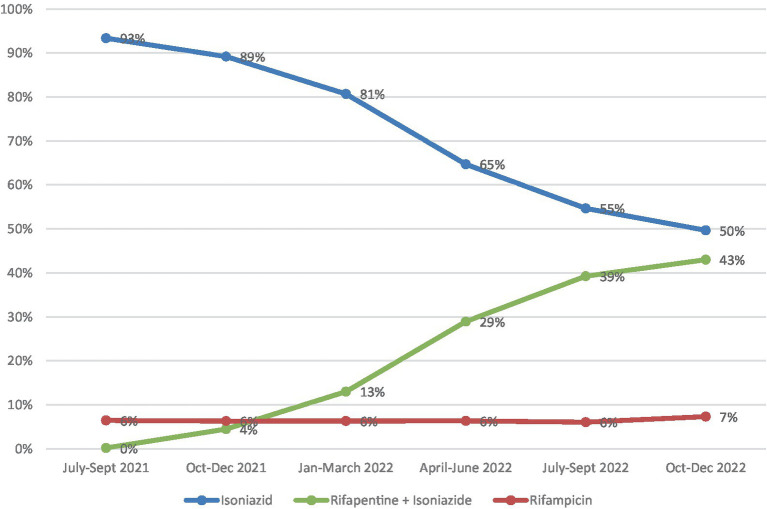
Number of TPT with different regimens initiated from July 2021 to December 2022, Brazil.

**Figure 5:**
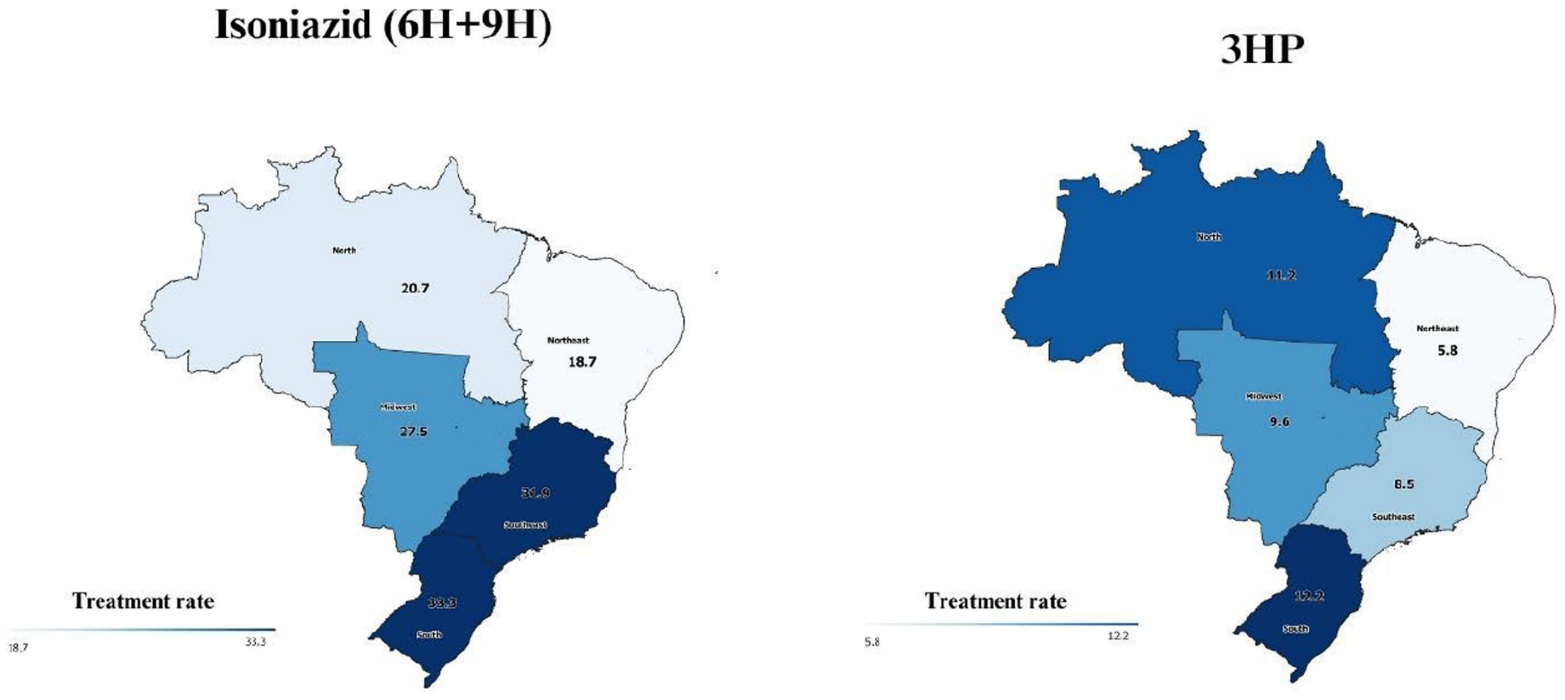
TPT with isoniazid (6H or 9H) and 3HP notified by Brazilian states from July 2021 to December 2022 (treatment rate per 100 cases of tuberculosis notified in SINAN in 2021/2022)*. *There is no data from the two states (Goiás in the Midwest and Santa Catarina in the South) that have not joined the IL-TB system.

The substitution of isoniazid (6H or 9H) by 3HP is progressing. The 3HP regimen represented less than 4% of the total administered by the end of 2021, reaching around 30% in the second half of 2022 and 40% in the last quarters of 2022. Rifampicin (4R) remained stable at around 6–7% (Figure 4). The ratio of TPT with isoniazid per 100 reported TB cases in Brazil were 25.8. The South and Southeast had the highest rates (33.3 and 31.9 respectively), and the Northeast the lowest (18.7). Considering 3HP, the rate for the country was 21.5. The regions with the highest rates were the South (12.2) and the North (11.2), and the lowest were the Northeast (5.8) and the Southeast (8.5) (Figure 5).

**Figure 6:**
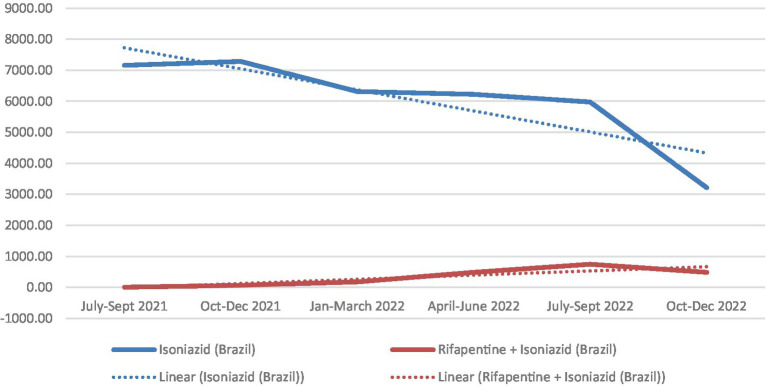
Total number of DDD per 1,000 inhabitants (DID) of Isoniazid for TPT per quarter. Brazil. July 2021 to December 2022 (treatments started). ^a^Estimated population 2021: 213.317.639; Population in 2022 (census): 203.062.512.

The number of DDD per 1,000 inhabitants per quarter confirmed the downward trend of isoniazid treatments (6H and 9H) and a slightly upward trend of the combined isoniazid treatment (3HP). As 3HP has a lower total dose per treatment (only 10,800 mg of isoniazid per complete 3HP treatment, compared to 81,000 or 54,000 for the 6H and 9H regimens respectively), the inclusion of 3HP has reduced the total consumption of isoniazid. Isoniazid consumption is still very high: even in the last quarter of 2022, when 3HP already accounted for 43% of treatments, the number of DDD of isoniazid from 6H or 9H was 6.7 times higher than that of 3HP isoniazid (Figure 6).

## 4 Discussion

TPT in asymptomatic contacts with TBI (positive TST) has been recommended in Brazil since 2010, but only in 2018, with the implementation of the IL-TB information system, observation of progress became possible. Our analysis of the progress shows a modest but steady increase in TPT prescription throughout the period (2018–2022). This increase occurred with small fluctuations over time. Additionally, after a small drop in the second quarter of 2020 due to the COVID-19 pandemic, the number of treatments prescribed increased steadily, with the exception of the last quarter, probably due to delay in reporting and system information uptake (17).

Implementation of 3HP shows a slow increase progressively replacing the regimen with isoniazid alone (6H or 9H). In 2022, the 3HP regimen became first-choice for TPT in the country. This replacement is desirable due to several factors already pointed out in the literature: the 3HP regimen has fewer side effects, especially less hepatotoxicity, and better therapeutic adherence compared to isoniazid alone (18, 19). Even lower rates of side effects are described in the literature with regimens in which isoniazid is absent, as is the case with the 4R regimen for TPT (4).

The fewer adverse effects of the 3HP regimen can be explained by the lower dose of isoniazid present in 3HP compared with the previous single-drug regimen. The impact in terms of drug consumption is not negligible: As Figure 6 shows, there is still substantial overexposure to isoniazid in the country. Although linear trends for DID show very different angular coefficients, 3HP is slowly and consistently rising while 6H + 9H is plummeting.

Although it was expected that the incorporation of a new technology (in this case, the 3HP regimen) would increase TPT due to the mobilization that it entails, such as training of health professionals and engagement of the scientific community, the increase in the total number of TPT prescription had already been observed since 2018 with the gradual expansion of IL-TB coverage, before the recommendation of the 3HP regimen.

Worldwide, contacts were the population with the lowest progress for TPT (8). Although they represent more than half of the indications for TPT in Brazil, they still represent only 16% of active TB index cases in Brazil notified in the same period. Considering that the average number household contacts is 3/index case (14), and that 30% of contacts are TST positive in the country (20), even considering losses of 10% in the steps of the cascade, we would expect 0.7 contacts per index case. Thus, there is a long way to go to expand TPT among contacts at a desirable rate in order to effectively reduce the incidence of tuberculosis in the population.

Figure 5 shows the number of TPT prescriptions per 100 active TB cases and regional differences can be perceived. In the South and the North 3HP has greater bearing in the treatment profile while in the Southeast 6H + 9H still prevails. This may reflect more attention of MoH in these regions (21).

The study presents some limitations. The IL-TB is a non-compulsory notification database, still in the process of being implemented in the country concurrently with the inclusion of the 3HP regimen. However, drug distribution depends on notification, so the database is reasonably complete. We also highlight the originality of this study as the first one to describe drug consumption using this data. The database was extracted in December 2022, which, knowingly due to the delay in information consolidation, may represent some inconsistency with the last quarter. The absence of a variable differentiating the 6H and 9H regimens for the calculation of the DDD, led us to estimate the proportion of these regimens using the valid cases of doses taken, resulting in an acceptable imputation.

Despite these limitations, we have not identified any other study describing the implementation of the 3HP regimen using secondary drug utilization data on a nationwide scale. Several experiences are described in the literature, mainly evaluating effectiveness, but always with a specific focus on certain programs (22), population groups (23, 24), cities and/or health units (25). All these studies found a high level of acceptance and completion of 3HP regimen.

Furthermore, the Brazilian experience is particular and interesting to analyze: since 1979, tuberculosis treatment has been entirely centralized at state level, and offered only through the Unified Health System, with a ban on the sale of anti-tuberculosis drugs in private drugstores. Our study therefore offers a rich panorama of the uptake of an essential technology by a national tuberculosis control program in a high-burden tuberculosis country setting (26).

The study points not only to the need to expand TPT in the country, but also to accelerate 3HP uptake and to encourage the municipalities to notify to the IL-TB system, since there is still a high level of underreporting. This obstacle is compounded by the resistance of the prescribing professionals themselves, who are still not used to recommending TPT for asymptomatic contacts. A broad policy of training the health network is needed to really consolidate TPT as a routine in health services.

An opportunity to reverse this situation is the Expand-TB project (27) being carried out by Rede-TB with funding from Stop TB. The project has been holding a series of training workshops and roundtables with professionals across the country with the aim of raising awareness among health services of the importance of expanding TPT as a strategy for eliminating TB, in line with the WHO’s sustainable development Goals for 2035. The rapid incorporation of 3HP will allow better adherence to TPT and protect the Brazilian population from overexposure to isoniazid.

## Data availability statement

The datasets presented in this article are not readily available because Brazilian law prohibits me from sending data that belongs to the Ministry of Health and that has been provided to me upon formal request and presentation of approval by an ethics committee. Requests to access the datasets should be directed to tuberculose@saude.gov.br.

## Ethics statement

The studies involving humans were approved by the Comitê de Ética em Pesquisa -CEP/ ENSP Fiocruz. The studies were conducted in accordance with the local legislation and institutional requirements. Written informed consent for participation was not required from the participants or the participants’ legal guardians/next of kin in accordance with the national legislation and institutional requirements.

## Author contributions

LM: Writing – original draft, Writing – review & editing. AT: Writing – original draft, Writing – review & editing. MRC: Writing – original draft, Writing – review & editing. MCC: Writing – review & editing. CO-d-C: Writing – original draft, Writing – review & editing.
